# Supplementary figures and images for: Enzymatic Antioxidant Defense System of Scots Pine Seedlings Under Conditions of Progressive Manganese Deficiency
Source: Biology (Basel). 2026 Jan 4;15(1):101. doi: 10.3390/biology15010101 (PMC12784764; doi:10.3390/biology15010101)

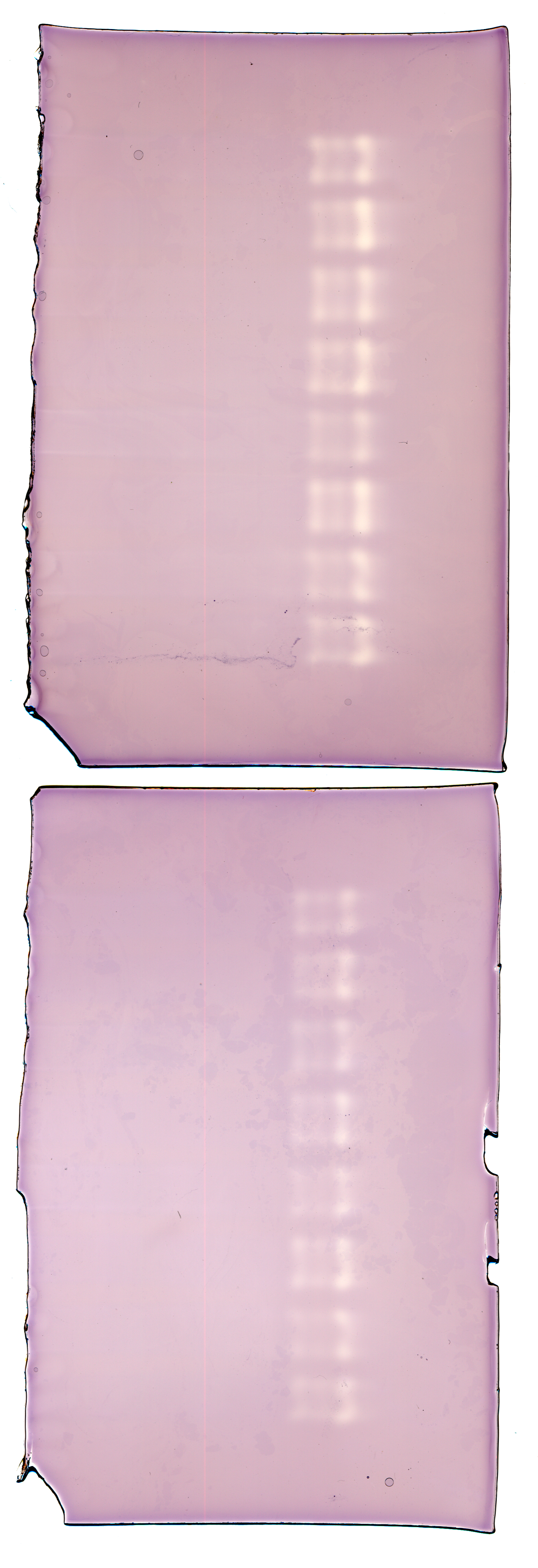

Supplement: Supplementary file 1 [file biology-15-00101-s001.zip › Figure S5. Source image for Figure 5A.tif]

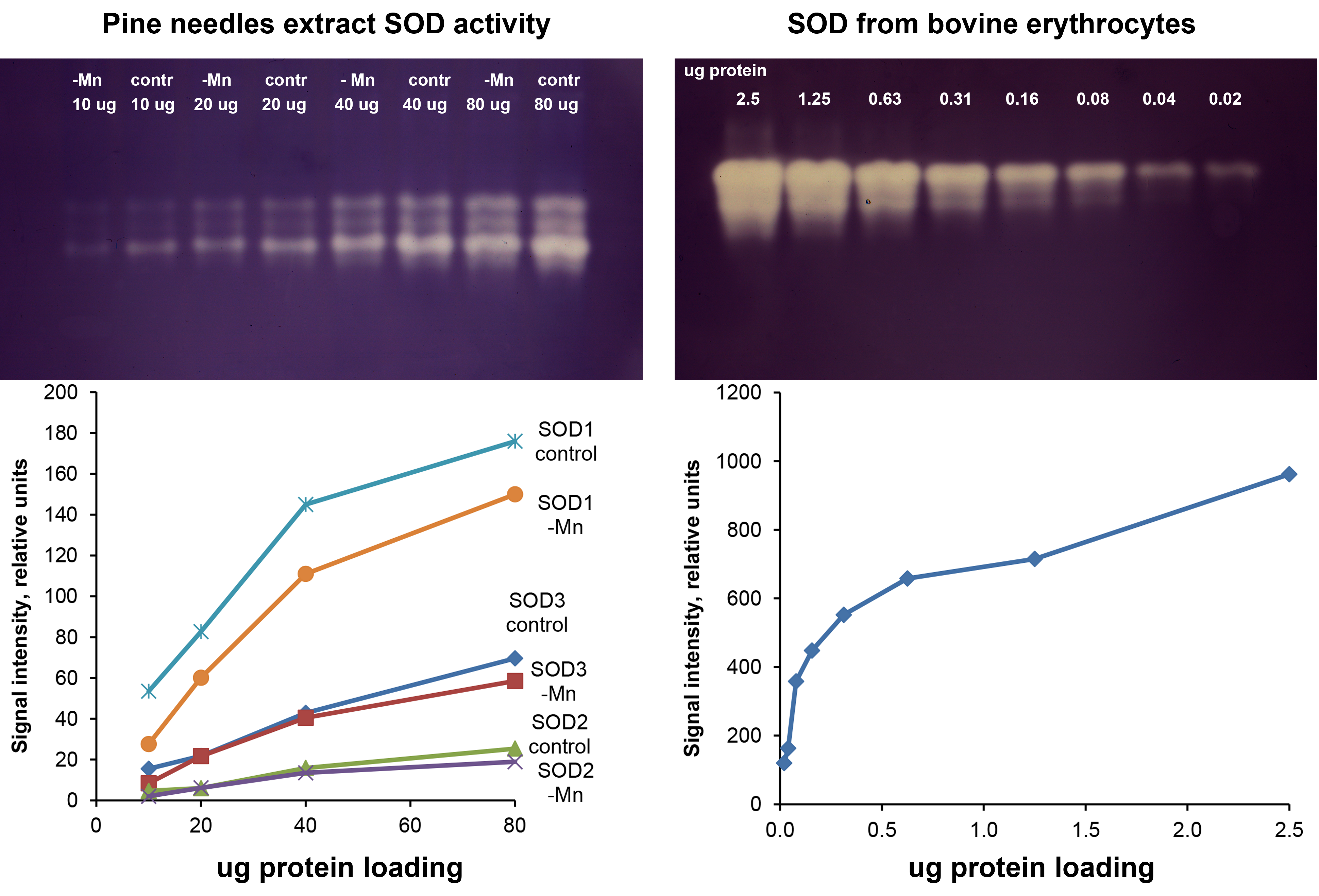

Supplement: Supplementary file 1 [file biology-15-00101-s001.zip › Figure S1.tif]

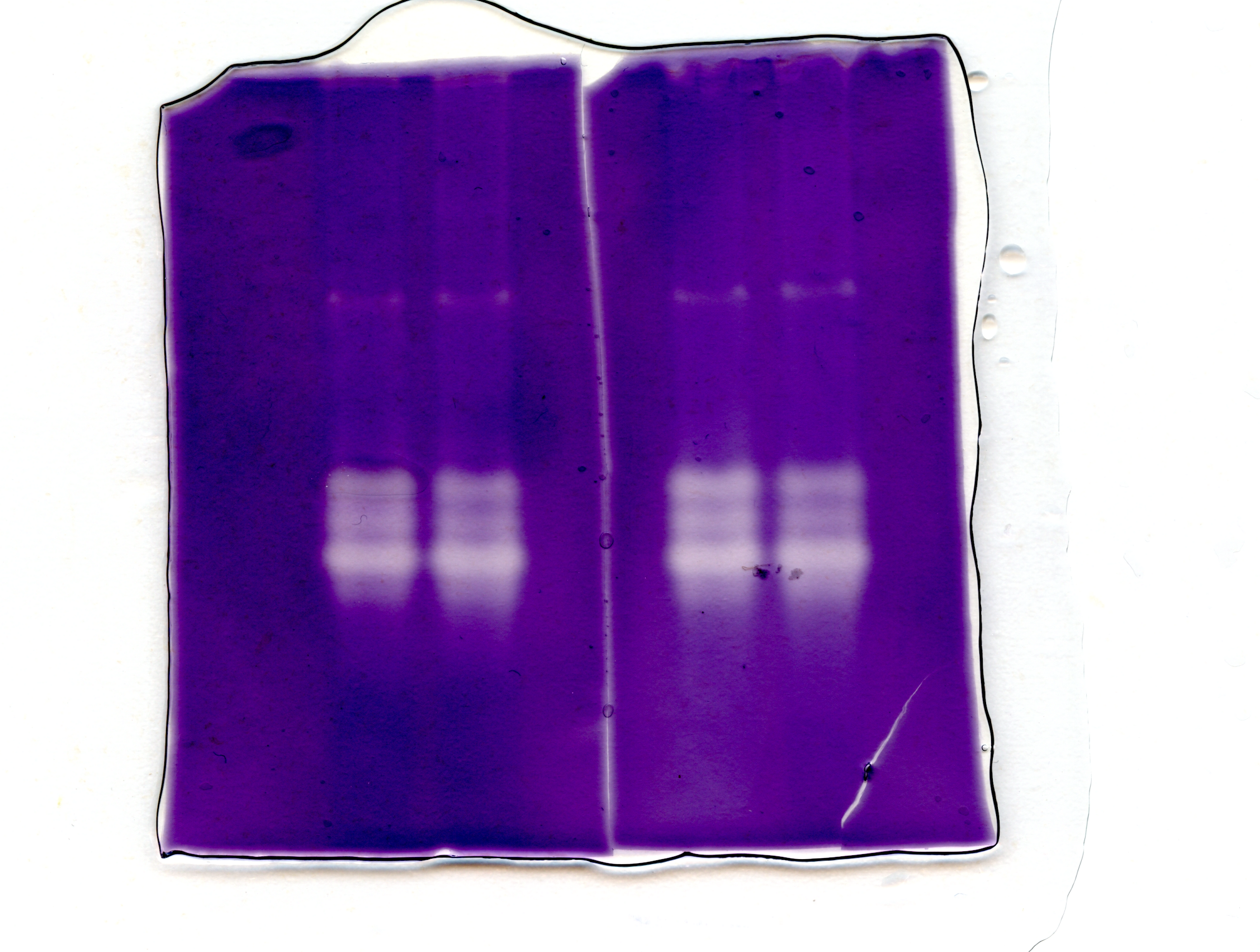

Supplement: Supplementary file 1 [file biology-15-00101-s001.zip › Figure S2. Source image for Figure 4A.tif]

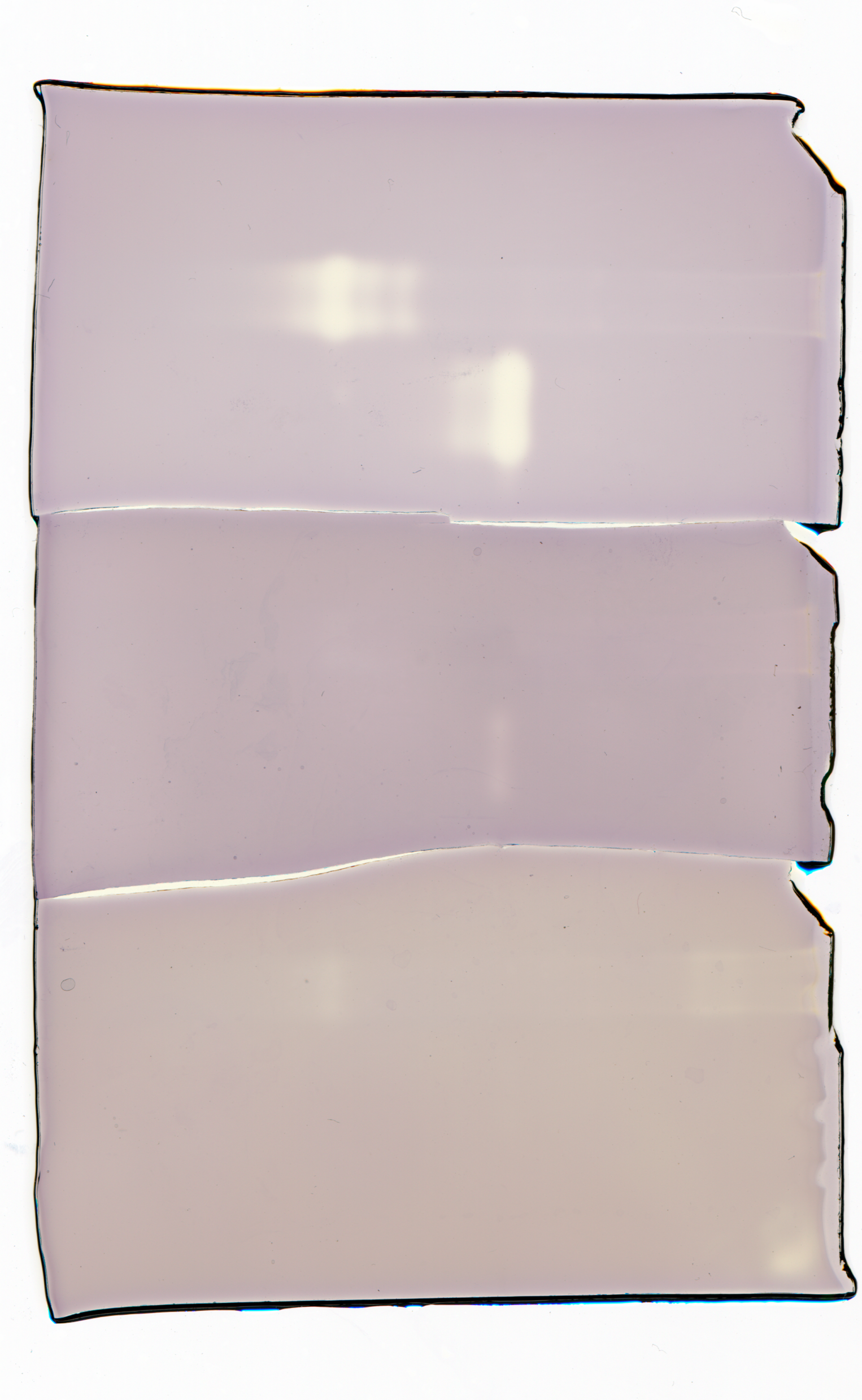

Supplement: Supplementary file 1 [file biology-15-00101-s001.zip › Figure S3. Source image for Figure 4B.tif]

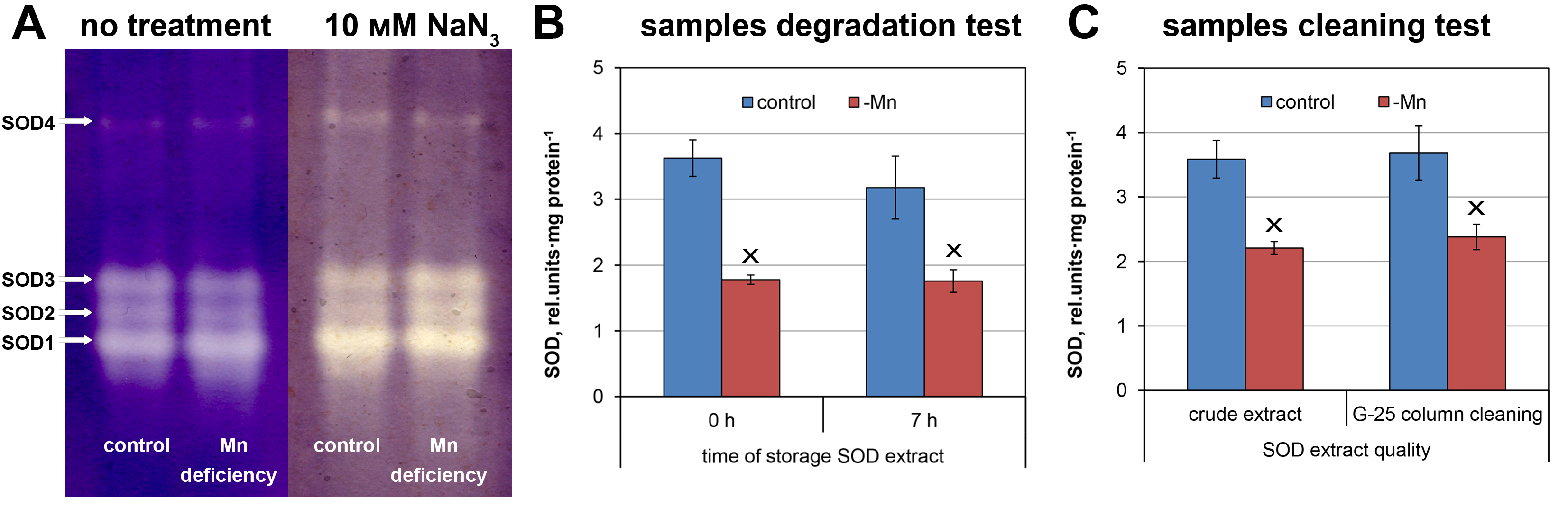

Supplement: Supplementary file 1 [file biology-15-00101-s001.zip › Figure S4_revised.tif]
